# Supplementary material for: Enthalpy efficiency of the soleus muscle contributes to improvements in running economy
Source: Proc Biol Sci. 2021 Jan 27;288(1943):20202784. doi: 10.1098/rspb.2020.2784 (PMC7893283; doi:10.1098/rspb.2020.2784)
Supplement: Methods Detailed information and figures [file rspb20202784supp1.docx]

***Title: Enthalpy efficiency of the soleus muscle contributes to improvements in running economy***

***Authors: Sebastian Bohm*, Falk Mersmann, Alessandro Santuz & Adamantios Arampatzis***

***Journal: Proceedings of the Royal Society B***

***DOI: 10.1098/rspb***

**Supplementary material 1: Methods section**

**Statistical power analysis**

A statistical power analysis was performed *a priori* to calculate the required sample size by means of the software G*Power (version 3.1.9.6, HHU Düsseldorf, Germany) [1]. For this purpose, we used the effect size of the rate of oxygen consumption from our previous intervention study with the same training regimen (d = 1.04) [2]. Since the main outcome of interest was the effect of training, the power analysis was conducted for the *post-hoc* time point comparison for the intervention group considering a Bonferroni correction of the p-values (α = 0.025 (adjusted), power 0.8, two-tailed paired t-test). The analysis revealed a required sample size of n = 12 for the intervention group.

**Exercise protocol**


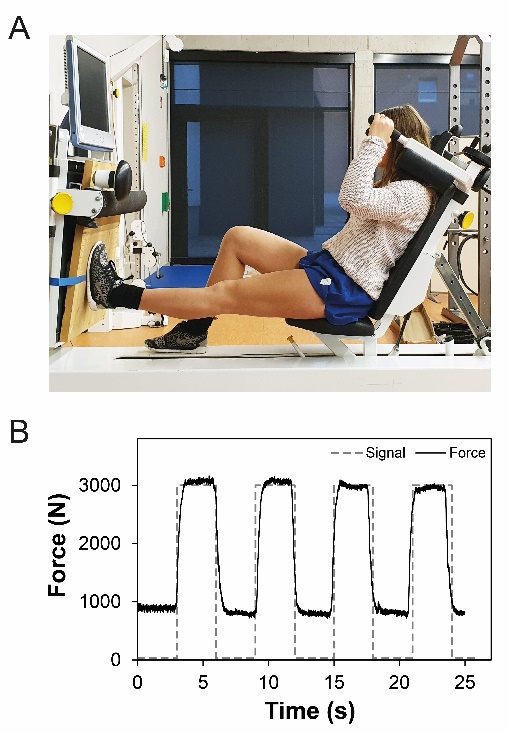


**Figure 1:** A conventional leg press was used for the muscle-tendon training of the m. triceps surae. The isometric plantar flexion contractions were performed at 5° dorsiflexion with the knee extended in a seating position (A). The leg press was instrumented with a force sensor in order to control the training stimulus by providing the participant with a visual feedback of the actual contraction intensity. The feedback curve was displayed together with the evidence-based loading profile defined by a sequence of 4 repetitions of 3 s loading and relaxation at 90% of the weekly-adjusted maximum voluntary plantar flexor strength in each of the 5 sets per session, 4 times a week (B).

**Strength of the plantar flexors and Achilles tendon stiffness**

The strength of the plantar flexors of the right leg was measured before and after the 14 weeks in a seated position (70° of hip flexion) with the knee extended using a Biodex dynamometer (Biodex Medical Inc., Syst.3, Shirley, NY, USA). Following a standardized warm-up, five maximum voluntary contractions (MVC) were performed in resting ankle joint angles of 10° plantar flexion to the individual maximum dorsiflexion angle (0° = foot perpendicular to shank) in equally distributed intervals (3-7°) to determine the maximum joint moment. The resultant ankle joint moment was calculated using an established inverse dynamics approach to account for misalignments between dynamometer and joint axis as well as passive and gravitational moments [3]. Furthermore, the contribution of the antagonistic muscles to the measured ankle joint moments in the different joint angles was considered by an previously reported EMG-based approach [4]. For this reason, the EMG activity of the antagonistic tibialis anterior muscle during the maximum plantar flexions was recorded. In separate trials, an individual relationship of EMG amplitude of the tibialis anterior muscle, agonistic moment as well as ankle joint angle was then established. Thereto, the EMG activity of tibialis anterior was measured at rest and during two submaximal isometric dorsiflexion contractions that displayed slightly lower and higher EMG magnitudes as during the maximum plantar flexions [4] in three different joint angles (i.e. dorsi flexion, neutral position and plantar flexion) within the assessed range of motion. The relationship was described by the regression equation $M_{coact}={EMG}_{tib. ant.} \cdot(a+b\cdot\alpha_{ankle}+c\cdot\alpha_{ankle}^{2})$, where *M_coact_* is the antagonistic joint moment during the maximum plantar flexion, *EMG_tib. ant_*_._ is the respective tibialis anterior EMG activity during the MVCs, *α_ankle_* the ankle joint angle measured via the Vicon system and *a*, *b* and *c* the individual regression coefficients. Thus, for each joint angle the relationship between moment and EMG activity was assumed to be linear because of the small differences of the EMG magnitude of the two submaximal isometric dorsal flexion contractions [4]. Further, the ankle joint angle-moment relationship presented by the three different measured angles was formulated by a quadratic function to account for the force-length dependence of the muscle [5]. The EMG activity of the tibialis anterior and soleus muscle was measured using a wireless EMG system (Myon m320RX, Myon AG, Baar, Switzerland) and two bipolar surface electrodes (2 cm inter-electrode distance) that were placed on the muscle at an acquisition frequency of 1000 Hz, synchronized with the kinematic data.

For the determination of Achilles tendon (AT) stiffness, five ramp-MVCs with steadily increasing effort from rest to maximum under the same considerations (i.e. accounting for axis misalignment, passive and gravitational moments and co-activation) were conducted at 0° ankle angle. The force applied to the AT was calculated as quotient of the joint moment and the individual tendon lever arm. The AT lever arm was determined for each participant by using the tendon excursion method [6,7]. In this method, the lever arm of the AT is calculated as the ratio of the m. gastrocnemius medialis myotendinous junction displacement obtained by ultrasonography at 25 Hz to the corresponding angular excursion of the ankle joint during a passive joint rotation by the dynamometer (5 °/s). The ratio was calculated over the interval of 5° dorsiflexion to 10° plantar flexion, where tendon deformation is negligible [8] and five passive rotation trials were averaged to ensure high reliability [9]. The lever arm values were further corrected for the alignment of the tendon occurring during contractions using the factor provided by Maganaris et al. (1998) [10]. The corresponding AT elongation during the ramp MVCs was analyzed based on the displacement of the gastrocnemius medialis-myotendinous junction (MTJ) visualized by B-mode ultrasonography captures (My Lab 60, Esaote, Genova, Italy, 25 Hz). ). The MTJ displacement artefacts due to an unavoidable increase in the plantar flexion angle during the MVCs were taken into account as they significantly affect the tendon elongation measurement [11]. For this reason, the MTJ displacement as a function of the ankle joint angle was analyzed in an additional trial where the ankle joint was passively rotated by the Biodex over the full range of motion at 5 °/s and then used to correct the angle-dependent displacements obtained during the MVCs. The force and elongation data of five ramp-MVCs were averaged to give a reliable measure of the AT elongation [9]. The AT stiffness was calculated between 50% and 100% of the maximum tendon force using linear regression [12]. In order to calculate AT strain, the rest length was measured from the tuberositas calcanei to the MTJ at an ankle angle of 20° (plantar flexed) and extended knee (i.e. a position that provides AT slackness [8]).

**Energetic cost of running**

During an 8-minute running trial on a treadmill (h/p cosmos mercury, Isny, Germany) at 2.5 m/s, a breath-by-breath cardio pulmonary exercise testing system (MetaLyzer 3B-R2, CORTEX Biophysik GmbH, Leipzig, Germany) recorded the percentage of concentration of oxygen and carbon dioxide expired. Oxygen consumption increases in the first two minutes of running and reaches a steady state plateau, therefore, the rate of oxygen consumption (V̇O_2_) and carbon dioxide production (V̇CO_2_) was calculated as average of the last three minutes [13], where a steady state level is ensured (fig. 1). Steady state was additionally visually confirmed by the rate of (V̇O2) for each individual and trial and a RER of <1.0 was controlled for during the post analysis. Running economy was expressed in units of energy by:

$$Energetic cost =16.89\cdot{\dot{V}O}_{2}+ 4.84\cdot{\dot{V}\mathrm{CO}}_{2}$$

where the energetic cost is presented in [W/kg] and V̇O_2_ and V̇CO_2_ in [ml/s/kg] [14,15]. To reduce test-retest variability [16], the shoes, time of testing and training activity (previous 72 hours) were the same for the pre and post measurements. No food intake was allowed during the last 3 hours before testing.


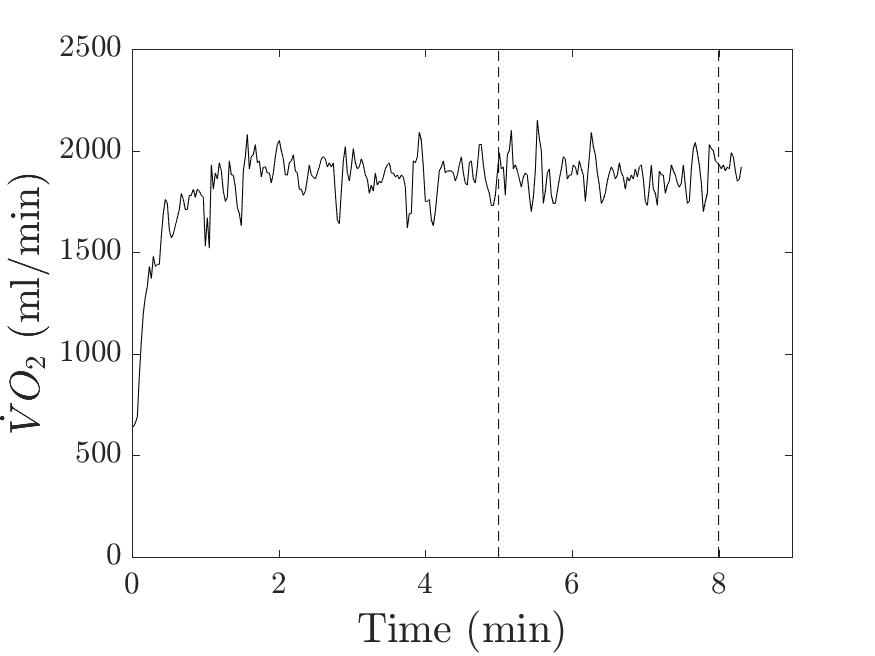


**Figure 2:** Example of the rate of oxygen consumption (V̇O2) of one representative participant. The two vertical lines indicate the interval during which the average rate of oxygen consumption was determined.

**Statistics**

An analysis of variance for repeated measures was performed for the plantar flexion moment (normalized to body mass) and AT stiffness (normalized to resting length) as well as metabolic energy cost, foot strike index and temporal gait characteristics during running with the time point as the within-subjects factor (pre vs. post) and the group as a between-subjects factor (intervention vs control). The *post-hoc* analysis was conducted separately for each group considering a Benjamini-Hochberg correction (adjusted p-values reported). Normality of the standardized residuals was controlled using the Kolmogorov-Smirnov test with Lilliefors correction.

Anthropometric group differences as well as baseline differences of the plantar flexion moment, AT stiffness and energetic cost were tested using a t-test for independent samples. A paired t-test was used to analyze the training effects on the assessed gait characteristics, kinematics and MTU and fascicle parameters. If normality tested by the Kolmogorov-Smirnov test was not given, the Wilcoxon signed rank test was applied. The level of significance was set to α = 0.05 and the statistical analyses were performed using SPSS (IBM Corp., version 22, NY, USA). Effect sizes (Hedges’ g to account for small sample sizes) in absolute values were calculated to assess the strength of the intervention effects, were 0.2 ≤ g < 0.5 indicate small, 0.5 ≤ g < 0.8 indicate medium, and g ≥ 0.8 indicate large effects [17].

**References**

1. Faul F, Erdfelder E, Lang A-G, Buchner A. 2007 G*Power 3: A flexible statistical power analysis program for the social, behavioral, and biomedical sciences. *Behavior Research Methods* **39**, 175–191. (doi:10.3758/BF03193146)

2. Albracht K, Arampatzis A. 2013 Exercise-induced changes in triceps surae tendon stiffness and muscle strength affect running economy in humans. *Eur J Appl Physiol* **113**, 1605–1615. (doi:10.1007/s00421-012-2585-4)

3. Arampatzis A, Morey-Klapsing G, Karamanidis K, DeMonte G, Stafilidis S, Brüggemann G-P. 2005 Differences between measured and resultant joint moments during isometric contractions at the ankle joint. *Journal of Biomechanics* **38**, 885–892. (doi:10.1016/j.jbiomech.2004.04.027)

4. Mademli L, Arampatzis A, Morey-Klapsing G, Brüggemann G-P. 2004 Effect of ankle joint position and electrode placement on the estimation of the antagonistic moment during maximal plantarflexion. *Journal of Electromyography and Kinesiology* **14**, 591–597. (doi:10.1016/j.jelekin.2004.03.006)

5. Bohm S, Mersmann F, Santuz A, Arampatzis A. 2019 The force–length–velocity potential of the human soleus muscle is related to the energetic cost of running. *Proceedings of the Royal Society B: Biological Sciences* **286**, 20192560. (doi:10.1098/rspb.2019.2560)

6. An KN, Takahashi K, Harrigan TP, Chao EY. 1984 Determination of muscle orientations and moment arms. *J Biomech Eng* **106**, 280–282.

7. Fath F, Blazevich AJ, Waugh CM, Miller SC, Korff T. 2010 Direct comparison of in vivo Achilles tendon moment arms obtained from ultrasound and MR scans. *J. Appl. Physiol.* **109**, 1644–1652. (doi:10.1152/japplphysiol.00656.2010)

8. De Monte G, Arampatzis A, Stogiannari C, Karamanidis K. 2006 In vivo motion transmission in the inactive gastrocnemius medialis muscle–tendon unit during ankle and knee joint rotation. *Journal of Electromyography and Kinesiology* **16**, 413–422. (doi:10.1016/j.jelekin.2005.10.001)

9. Schulze F, Mersmann F, Bohm S, Arampatzis A. 2012 A wide number of trials is required to achieve acceptable reliability for measurement patellar tendon elongation in vivo. *Gait Posture* **35**, 334–338. (doi:10.1016/j.gaitpost.2011.09.107)

10. Maganaris CN, Baltzopoulos V, Sargeant AJ. 1998 Changes in Achilles tendon moment arm from rest to maximum isometric plantarflexion: in vivo observations in man. *The Journal of Physiology* **510**, 977–985. (doi:10.1111/j.1469-7793.1998.977bj.x)

11. Arampatzis A, Monte GD, Karamanidis K. 2008 Effect of joint rotation correction when measuring elongation of the gastrocnemius medialis tendon and aponeurosis. *J Electromyogr Kinesiol* **18**, 503–508. (doi:10.1016/j.jelekin.2006.12.002)

12. Arampatzis A, Karamanidis K, Albracht K. 2007 Adaptational responses of the human Achilles tendon by modulation of the applied cyclic strain magnitude. *J. Exp. Biol* **210**, 2743–2753. (doi:10.1242/jeb.003814)

13. Albracht K, Arampatzis A. 2013 Exercise-induced changes in triceps surae tendon stiffness and muscle strength affect running economy in humans. *European Journal of Applied Physiology* **113**, 1605–1615. (doi:10.1007/s00421-012-2585-4)

14. Kipp S, Byrnes WC, Kram R. 2018 Calculating metabolic energy expenditure across a wide range of exercise intensities: the equation matters. *Appl Physiol Nutr Metab* **43**, 639–642. (doi:10.1139/apnm-2017-0781)

15. Péronnet F, Massicotte D. 1991 Table of nonprotein respiratory quotient: an update. *Canadian Journal of Sport Sciences* **16**, 23–9.

16. Saunders PU, Pyne DB, Telford RD, Hawley JA. 2004 Factors Affecting Running Economy in Trained Distance Runners. *Sports Med* **34**, 465–485. (doi:10.2165/00007256-200434070-00005)

17. Cohen J. 1988 *Statistical Power Analysis for the Behavioral Sciences*. Psychology Press.
