# Supplementary material for: Enthalpy efficiency of the soleus muscle contributes to improvements in running economy
Source: Proc Biol Sci. 2021 Jan 27;288(1943):20202784. doi: 10.1098/rspb.2020.2784 (PMC7893283; doi:10.1098/rspb.2020.2784)
Supplement: Discussion Sensitivity analysis data table [file rspb20202784supp2.docx]

***Title: Enthalpy efficiency of the soleus muscle contributes to improvements in running economy***

***Authors: Sebastian Bohm*, Falk Mersmann, Alessandro Santuz & Adamantios Arampatzis***

***Journal: Proceedings of the Royal Society B***

***DOI: 10.1098/rspb***

**Supplementary material 2: Discussion section**

**Sensitivity analysis of V_max_**

**Table 1:** Enthalpy efficiency (mean ± SD) of the soleus muscle during running before and after the training intervention based on the data given by Hill (1964) [1] and Barclay et al. (1993) [2] for different values of V_max_ (n = 13) and tested by a t-test for depended samples (two-tailed).

|  | **Hill (1964)** | | | **Barclay et al. (1993)** | | |
| --- | --- | --- | --- | --- | --- | --- |
|  | **pre** | **post** | **p-value** | **pre** | **post** | **p-value** |
| **Stance phase** |  |  |  |  |  |  |
| V_max_ | 0.386 ± 0.029 | 0.409 ± 0.027 | 0.025 | 0.251 ± 0.025 | 0.270 ± 0.024 | 0.039 |
| V_max-10%_ | 0.390 ± 0.026 | 0.414 ± 0.414 | 0.013 | 0.254 ± 0.022 | 0.274 ± 0.021 | 0.014 |
| V_max-20%_ | 0.394 ± 0.024 | 0.418 ± 0.020 | 0.006 | 0.256 ± 0.020 | 0.278 ± 0.018 | 0.004 |
| V_max-30%_ | 0.396 ± 0.022 | 0.420 ± 0.020 | 0.003 | 0.257 ± 0.018 | 0.280 ± 0.014 | 0.002 |
|  |  |  |  |  |  |  |
| **MTU lengthening** |  |  |  |  |  |  |
| V_max_ | 0.347 ± 0.057 | 0.398 ± 0.045 | 0.006 | 0.218 ± 0.047 | 0.261 ± 0.040 | 0.007 |
| V_max-10%_ | 0.356 ± 0.053 | 0.403 ± 0.040 | 0.006 | 0.225 ± 0.043 | 0.266 ± 0.035 | 0.005 |
| V_max-20%_ | 0.365 ± 0.048 | 0.407 ± 0.034 | 0.005 | 0.232 ± 0.039 | 0.269 ± 0.030 | 0.004 |
| V_max-30%_ | 0.373 ± 0.043 | 0.410 ± 0.029 | 0.006 | 0.239 ± 0.034 | 0.270 ± 0.024 | 0.006 |

**References**

1. Hill AV. 1964 The effect of load on the heat of shortening of muscle. *Proceedings of the Royal Society of London. Series B. Biological Sciences* **159**, 297–318. (doi:10.1098/rspb.1964.0004)

2. Barclay CJ, Constable JK, Gibbs CL. 1993 Energetics of fast- and slow-twitch muscles of the mouse. *The Journal of Physiology* **472**, 61–80. (doi:10.1113/jphysiol.1993.sp019937)
